# Re-Entrant Honeycomb Auxetic Structure with Enhanced Directional Properties

**DOI:** 10.3390/ma15228022

**Published:** 2022-11-14

**Authors:** Farrukh Mustahsan, Sohaib Z. Khan, Asad A. Zaidi, Yaser H. Alahmadi, Essam R. I. Mahmoud, Hamad Almohamadi

**Affiliations:** 1 College of Electrical & Mechanical Engineering, National University of Sciences and Technology, Rawalpindi 43701, Pakistan; 2 Department of Mechanical Engineering, Faculty of Engineering, Islamic University of Madinah, Madinah 42351, Saudi Arabia; 3 Department of Mechanical Engineering, Faculty of Engineering Science and Technology, Hamdard University, Karachi 74600, Pakistan; 4 Department of Chemical Engineering, Faculty of Engineering, Islamic University of Madinah, Madinah 42351, Saudi Arabia

**Keywords:** negative Poisson’s ratio, auxetic material, re-entrant honeycomb, tensile loading, Young’s modulus

## Abstract

This paper presents a modified re-entrant honeycomb auxetic structure. The structure is constructed by adding an additional horizontal member between the vertical and re-entrant member of the semi-re-entrant honeycomb model to increase the overall compliance of the structure in order to obtain higher values of negative Poisson’s ratio (NPR). An analytical model of the structure is presented, taking into account the bending, shear, and axial deformations. The model is verified using finite element analysis (FEA) and tensile testing. The results of FEA and tensile testing corroborate the results of the presented mathematical model. The structure is also compared to the existing re-entrant honeycomb structure. The newly added strut has shown a direct effect on the directional properties of the overall structure. With an increase in the newly added strut to re-entrant lengths, NPR was significantly enhanced in the x-direction and reduced in the y-direction loadings. The structure shows an improved Young’s modulus compared to solid material in both loading directions, especially for the low values of the new strut and re-entrant lengths ratio. The structure also shows that high NPR can be achieved for low relative density compared to semi re-entrant honeycomb structure.

## 1. Introduction

One of the most important properties of a material is its Poisson’s ratio. Most materials possess a positive Poisson’s ratio, meaning that when the material is stretched in one direction, its cross-section decreases in the transverse direction. However, a particular class of materials exhibits a negative Poisson’s ratio (NPR), meaning that their cross-section will increase in the transverse direction when a tensile load is applied. Such materials/structures are also commonly known as auxetic or NPR materials/structures. Auxetic materials provide increased shear modulus, fracture toughness, in-plain strain fracture resistance, energy absorption, indentation resistance, and acoustic response [1,2,3,4,5,6].

A study by Donoghue et al. [7] found that auxetic composite laminates require more energy for a crack to propagate than a conventional laminate under tensile loading, indicating that auxetic composites provide better fracture toughness than conventional composites. Another study by Choi and Lakes [8] compared the fracture stiffness of auxetic foams with conventional foams and observed that auxetic foams provide greater fracture toughness than conventional ones even with a smaller Young’s modulus. Yao et al. [9] proposed and tested an anti-pullout auxetic pedicle screw made using the re-entrant honeycomb structure and found that the greater auxeticity of the screw results in a better anti-pullout performance, which they proposed can resolve the loosening and pulling out issues of typical pedicle screws currently being used. Several studies were found in the literature on the behavior of auxetic structures under impact loading. A study by Zhang et al. [4] showed that sandwich panels with re-entrant honeycomb cores absorbed explosive loads better than the honeycomb structure.

There have been numerous studies on different types of auxetic unit cells in the literature, especially in the past decade, due to the ease of manufacturing such structures because of advancements in additive manufacturing technologies. The traditional periodic cellular structure was proposed by Gibson et al. [10] in the form of a 2D re-entrant honeycomb. The structure is made by inverting the direction of inclined members in a typical honeycomb structure. The resultant structure provides NPR in both the x- and y-directions. A few years later, Gibson et al. [10] provided a mathematical model for the 2D re-entrant model to calculate its Poisson’s ratio and Young’s modulus based on the flexural method. Subsequently, Masters et al. [11] improved on this by developing a model for these elastic constants with the inclusion of stretching, hinging, and flexure. The advent of additive manufacturing gave way to the manufacturing of three-dimensional versions of the auxetic structures, which were difficult to manufacture using conventional means; one such structure was proposed by Lang et al. [12], who provided a model for a 3D version of the re-entrant honeycomb model and compared it with FEA and experimental testing results.

The re-entrant honeycomb model is the most widely researched auxetic structure [13,14,15,16,17,18]. Re-entrant is used to describe something directed inwards or that has a negative angle. The idea of an auxetic structure was first proposed by Gibson et al. [10] in 1982 in the form of a 2D re-entrant honeycomb by introducing a negative angle in the inclined member of a typical honeycomb structure. Recently, Al-Rifaie et al. [19] presented their patented idea of using a re-entrant honeycomb structure manufactured using aluminum sheets by bending in different orientations, and the structure showed good damping properties.

Multiple structure variations have been proposed to enhance or modify the mechanical properties of this structure. These structures are made by either adding additional members to the existing structure or combining it with other types of auxetic structures. One such structure was proposed by Sigmund et al. [19], where they used topology optimization techniques to devise a variation of the re-entrant honeycomb model. Fu et al. [20] proposed a structure created by the additional embedding of a rhombic configuration into a re-entrant honeycomb model. The structure results in enhanced buckling strength and in−plane stiffness. Another such structure was proposed by Harkati et al. [21] by adding a chamfer to the edges of the diagonal member. An analytical model of the structure was presented, supported by finite element analysis (FEA) results. Adding the chamfer increases NPR behavior. Bezazi et al. [22] presented a mathematical model for a novel central symmetric structure by adding a chamfer to the existing honeycomb structure. The authors only considered bending deflection in the model, calculated the strain in the x-direction caused by loading in the respective direction, did the same for the y-direction, and divided them both to obtain the Poisson’s ratio, because of the nature of the central symmetric model. This approach may not be applicable for other unit cells to calculate values of Poisson’s ratio, especially in the case of non−isotropic structures. Zhu et al. [23] introduced a modified version of the re-entrant structure by introducing zigzag inclined members in the re-entrant honeycomb structure resulting in an increase in stiffness and auxeticity in one of the principal directions. Wu et al. [24] combined the re-entrant honeycomb and regular honeycomb structure to create a hybrid structure that can provide positive, negative, and zero Poisson’s ratio depending on its configuration. Zhang et al. [25] studied the energy absorption properties of aluminum composite tubes with hexagonal honeycomb and re-entrant honeycomb fillings and found that the one with the auxetic filling exhibits superior ductility compared to its non-auxetic counterpart. Nguyen et al. [6] analyzed a sandwich panel with a re-entrant honeycomb core and graphene nanoplates as the skin, and such a structure can benefit from the energy absorption and lightweight nature of the structure.

A periodic cellular structure is proposed in this paper by adding an extra member to the existing re-entrant honeycomb structure.

An additional strut is introduced in the existing re-entrant honeycomb structure to enhance the auxetic behavior of the structure. A complete analytical model is introduced to cater to in−plane bending, shear, and axial deformation. Variation in the newly introduced strut length is analyzed analytically, using FEA, and experimentally. A detailed analysis of the proposed structure is conducted by varying the unit cell parameters through the analytical equations and FEA.

## 2. Unit Cell Design

**Figure 1:**
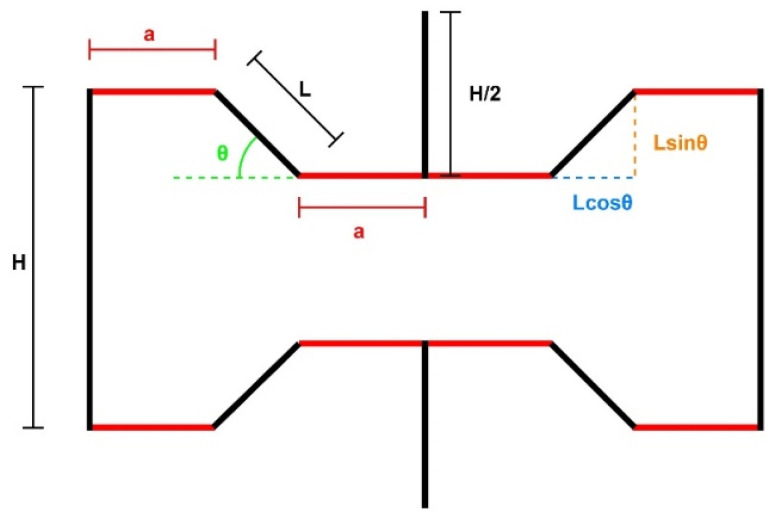
Unit cell of the proposed structure.

The proposed unit cell is shown in Figure 1. Horizontal members have been added to both sides between the re-entrant part and the vertical members of the re-entrant honeycomb structure. The newly added members (shown in red) will increase the compliance of the structure, which should result in increased deflections, especially in the y-direction, which should increase the NPR of the structure. The length of these members is the same and is denoted by a. The structure can be defined entirely using six perimeters which are: the length of the inclined member L, the length of the vertical member H, the length of the newly introduced member a, the thickness of the struts t, the depth of the structure in out-of-plane direction b, and the angle made by the inclined member with the horizontal axis θ.

The total length of the structure in the **x-** and **y-direction** can be calculated as follows:

(1) $$l_{x}=2\left(2a+Lcos\theta \right)$$

(2) $$l_{y}=2\left(H-Lsin\theta \right)$$

Here $l_{x}$ is the length in the **x-direction** and $l_{y}$ is the length in the **y-direction**. If we are to fit the structure in a cuboid, the volume $V_{s}$ that the structure will occupy can be calculated as:

(3) $$V_{s}=bl_{x}l_{y}=4b\left(2a+Lcos\theta \right)\left(H-Lcos\theta \right)$$

The actual volume $V_{a}$ occupied by the structure can be calculated by adding the individual volumes of every member, which comes out to be:

(4) $$V_{a}=2bt\left(H+4a+2L\right)$$

If we consider the structure as it is or model it as a cuboid with the original structure’s properties, the model’s mass ***m*** will remain the same. The relative density is defined as the ratio of the cuboid model’s density with the model’s actual density. Hence the relative density of the structure can be calculated as:

(5) $$\frac{\rho_{s}}{\rho_{a}}=\frac{m}{V_{s}}\times \frac{V_{a}}{m}=\frac{t\left(H+4a+2L\right)}{2\left(2a+Lcos\theta \right)\left(H-Lsin\theta \right)}$$

The parameters can be normalized by taking the ratios with a fixed parameter. This makes an analysis of the structure much more straightforward. For our case, the fixed parameter is the length of the inclined member ***L***. After inserting the normalized parameters, Equation (5) becomes.

(6) $$\frac{\rho_{s}}{\rho_{a}}=\left(\frac{t}{L}\right)\frac{\left(\frac{H}{L}+\frac{4a}{L}+2\right)}{2\left(\frac{2a}{L}+cos\theta \right)\left(\frac{H}{L}-sin\theta \right)}$$

Because we are adding an additional member to an existing structure, making the value of a equal to zero yields the relative density of the re-entrant honeycomb model.

## 3. Analytical Model

Simple mechanics of materials techniques can be used to calculate the elastic parameters of the structure. By looking at the structure in Figure 1, it can be seen that the structure is symmetric along the **X** and **Y** axes. Hence the elastic parameters can be calculated using a quarter model since the strain of the structure will remain the same in both instances because of the differences in lengths. The Poisson’s ratio and Young’s modulus in the x-direction are denoted by $v_{x}$ and $E_{x}$, respectively, and for the y-direction by $v_{y}$ and $E_{y}$**.** The shear modulus is denoted by G. It is assumed that the structure is located in the middle of the lattice to avoid boundary effects. Three types of deformations are considered for the model; these include flexural/bending deformations, shear deformations, and axial deformations. The structure is first loaded in both principal axes, one at a time, to calculate the elastic parameters in the respective direction, then a shear load is applied to calculate the shear modulus of the structure.

### 3.1. X-Direction

**Figure 2:**
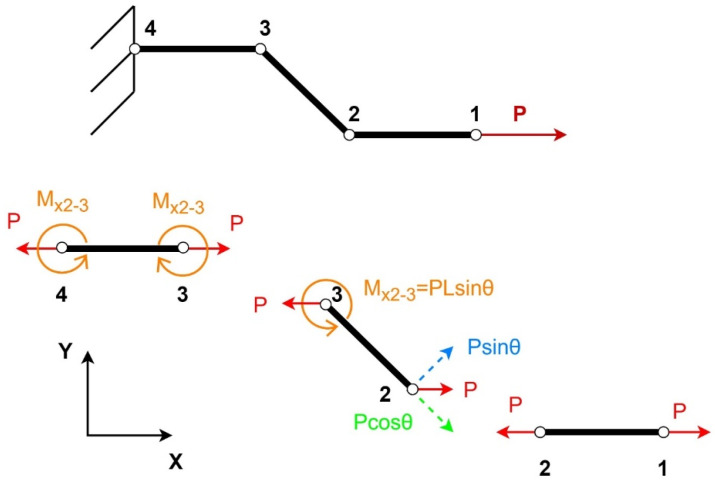
Free body diagram for loading in the x-direction.

The free body diagram (FBD) of the structure under loading in the x-direction is given in Figure 2.

The bending deflection of the re-entrant member consists of the bending caused by the force P and the deflection caused as a result of the moment induced in the horizontal member, and it can be calculated as [26]:

(7) $$\delta_{fX}=\frac{PL^{3}sin\theta }{12E_{s}I}\left(6\frac{a}{L}+1\right)$$

The bending deflection caused due to moment M on member 2–3 is:

(8) $$\delta_{faX}=\frac{PLa^{2}sin\theta }{2E_{s}I}$$

The shear deflection $\delta_{six}$ caused in the inclined member can be calculated by [10]:

(9) $$\delta_{siX}=\frac{PL^{3}sin\theta }{12E_{s}I}\left(2.4+1.5v_{s}\right){\left(\frac{t}{L}\right)}^{2}$$

The axial deformation of the inclined member can be calculated as follows [27]:

(10) $$\delta_{aiX}=\frac{PLcos\theta }{E_{s}bt}$$

Similarly, the deformations for the horizontal members can be calculated by [27]:

(11) $$\delta_{ahX}=\frac{2Pa}{E_{s}bt}$$

The total deflection $\left(\delta_{xx}\right)$ of the structure in the **x-direction** equals:

(12) $$\delta_{xx}=\delta_{fx}sin\theta +\delta_{six}sin\theta +\delta_{aix}cos\theta +\delta_{ahx}$$

After simplifying, the equation becomes

(13) $$\delta_{xx}=\frac{PL^{3}sin\theta^{2}}{12E_{s}I}\left(1+6\frac{a}{L}+\left(2.4+1.5v_{s}+\frac{1}{tan\theta^{2}}+\frac{1}{sin\theta^{2}}\frac{a}{L}\right){\left(\frac{t}{L}\right)}^{2}\right)$$

The total deflection $\left(\delta_{xy}\right)$ of the structure in the **y-direction** equals:

(14) $$\delta_{xy}=\delta_{fx}cos\theta +\delta_{six}cos\theta +\delta_{faX}$$

(15) $$\delta_{xy}=\frac{PL^{3}sin\theta cos\theta }{12E_{s}I}\left(1+6\frac{a}{L}+\left(2.4+1.5v_{s}\right){\left(\frac{t}{L}\right)}^{2}+\frac{1}{cos\theta }{\left(\frac{a}{L}\right)}^{2}\right)$$

Strain is defined as the ratio of the deflections of a material to its un-deformed length. Hence the strain $\epsilon_{xx}$ in the **x-direction** becomes [28]:

(16) $$\epsilon_{xx}=\frac{\delta_{xx}}{2a+Lcos\theta }$$

(17) $$\epsilon_{xx}=\frac{PL^{2}sin\theta^{2}}{12E_{s}I\left(2\frac{a}{L}+cos\theta \right)}\left[1+6\frac{a}{L}+\left(2.4+1.5v_{s}+\frac{1}{tan\theta^{2}}+\frac{1}{sin\theta^{2}}\frac{a}{L}\right){\left(\frac{t}{L}\right)}^{2}\right]$$

Similarly, the strain $\epsilon_{xy}$ in the y-direction [28]:

(18) $$\epsilon_{xy}=\frac{\delta_{xy}}{H-Lsin\theta }$$

(19) $$\epsilon_{xy}=\frac{PL^{2}sin\theta cos\theta }{12E_{s}I\left(\frac{H}{L}-sin\theta \right)}\left[1+6\frac{a}{L}+\left(2.4+1.5v_{s}\right){\left(\frac{t}{L}\right)}^{2}+\frac{1}{cos\theta }{\left(\frac{a}{L}\right)}^{2}\right]$$

The Poisson’s ratio is the ratio of lateral direction strain to the strain in the direction of the applied force. Hence the Poisson’s ratio for loading in the **x-direction** equals [28]:

(20) $$v_{x}=-\frac{\epsilon_{xy}}{\epsilon_{xx}}$$

(21) $$v_{x}=-\frac{\left(\frac{2a}{L}+cos\theta \right)cos\theta }{\left(\frac{H}{L}-sin\theta \right)sin\theta }\left\{\frac{\left[1+6\frac{a}{L}+\left(2.4+1.5v_{s}\right){\left(\frac{t}{L}\right)}^{2}+\frac{1}{cos\theta }{\left(\frac{a}{L}\right)}^{2}\right]}{\left[1+6\frac{a}{L}+\left(2.4+1.5v_{s}+\frac{1}{tan\theta^{2}}+\frac{1}{sin\theta^{2}}\frac{a}{L}\right){\left(\frac{t}{L}\right)}^{2}\right]}\right\}$$

The Young’s modulus for loading in the **x-direction** is defined as [28]:

(22) $$E_{x}=\frac{\sigma_{xx}}{\varepsilon_{yy}}$$

(23) $$E_{x}=E_{s}{\left(\frac{t}{L}\right)}^{3}\frac{\left(\frac{2a}{L}+cos\theta \right)}{\left(\frac{H}{L}-sin\theta \right)sin^{2}\theta }\left\{\frac{1}{\left[1+6\frac{a}{L}+\left(2.4+1.5v_{s}+\frac{1}{tan\theta^{2}}+\frac{1}{sin\theta^{2}}\frac{a}{L}\right){\left(\frac{t}{L}\right)}^{2}\right]}\right\}$$

### 3.2. Y-Direction

**Figure 3:**
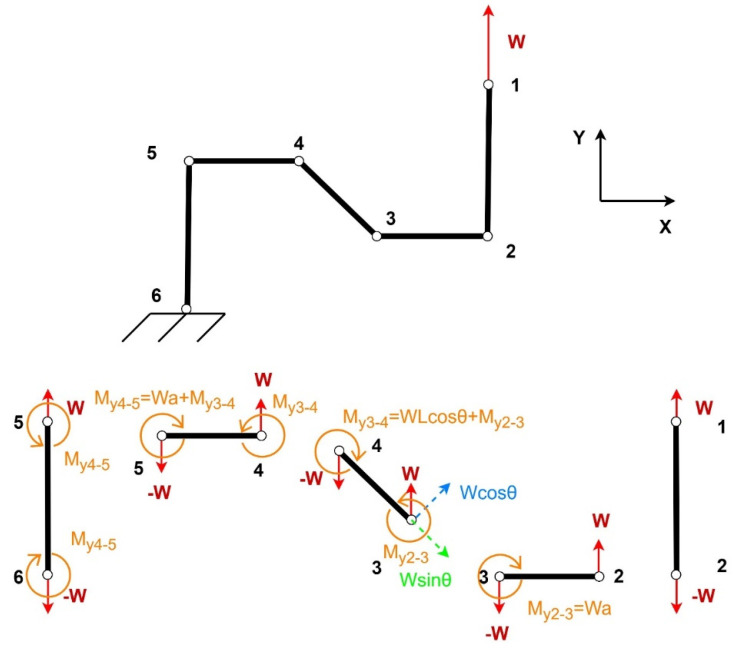
Free body diagram for loading in the y-direction.

The FBD of a quarter structure under loading in the y-direction is given in Figure 3.

The bending deflection caused in the re-entrant member can be calculated as [28]

(24) $$\delta_{rY}=\frac{WL^{3}cos\theta }{12E_{s}I}$$

The deflection caused as a result of moment M_y2-3_ equals [28]

(25) $$\delta_{rmY}=\frac{WaL^{2}cos\theta }{2E_{s}I}$$

The deflection of the horizontal member 4–5 is

(26) $$\delta_{hmY}=\frac{Wa^{3}}{2E_{s}I}+\frac{Wa^{2}Lcos\theta }{2E_{s}I}$$

The shear deflection of the structure can be calculated as [10]:

(27) $$\delta_{srY}=\frac{WL^{3}cos\theta }{12E_{s}I}\left(2.4+1.5v_{s}\right){\left(\frac{t}{L}\right)}^{2}$$

Moreover, the axial deformation of the re-entrant member can be calculated by [28]

(28) $$\delta_{arY}=\frac{WLsin\theta }{E_{s}bt}$$

Similarly, the axial deformation in the vertical members can be calculated by [28]

(29) $$\delta_{avY}=\frac{WH}{E_{s}bt}$$

The total deflection of the horizontal members can be calculated as follows [28]:

(30) $$\delta_{hY}=\frac{Wa^{3}}{6E_{s}I}$$

The total deformation in the **y-direction** can be calculated by

(31) $$\delta_{yy}=\delta_{rY}cos\theta +\delta_{srY}cos\theta +\delta_{arY}sin\theta +\delta_{rmY}cos\theta +\delta_{hmY}+\delta_{avY}$$

After simplification, we get

(32) $$\delta_{yy}=\frac{PL^{3}cos^{2}\theta }{12E_{s}I}\left[1+6\frac{a}{L}+6{\left(\frac{a}{L}\right)}^{2}\frac{1}{cos\theta }+8{\left(\frac{a}{L}\right)}^{3}\frac{1}{cos^{2}\theta }+\left(2.4+1.5v_{s}+\frac{1}{\tan^{2}\theta }+\frac{H}{L}\frac{1}{cos^{2}\theta }\right){\left(\frac{t}{L}\right)}^{2}\right]$$

Similarly, for deflections in the x-direction, we get

(33) $$\delta_{yx}=\frac{PL^{3}sin\theta cos\theta }{12E_{s}I}\left[1+6\frac{a}{L}+2{\left(\frac{a}{L}\right)}^{2}\frac{1}{cos\theta }+2{\left(\frac{a}{L}\right)}^{3}\frac{1}{sin\theta cos\theta }+\left(2.4+1.5v_{s}\right){\left(\frac{t}{L}\right)}^{2}\right]$$

The strain $\epsilon_{yy}$ in the **y-direction** equals

(34) $$\epsilon_{yy}=\frac{\delta_{yy}}{H-Lsin\theta }$$

(35) $$\epsilon_{yy}=\frac{F_{2}L^{3}cos\theta^{2}}{12E_{s}I\left(H-Lsin\theta \right)}\left[1+6\frac{a}{L}+6{\left(\frac{a}{L}\right)}^{2}\frac{1}{cos\theta }+8{\left(\frac{a}{L}\right)}^{3}\frac{1}{cos^{2}\theta }+\left(2.4+1.5v_{s}+\frac{1}{\tan^{2}\theta }+\frac{H}{L}\frac{1}{cos^{2}\theta }\right){\left(\frac{t}{L}\right)}^{2}\right]$$

The strain $\epsilon_{yx}$ in the **x-direction** is

(36) $$\epsilon_{yx}=\frac{\delta_{yx}}{2a+Lcos\theta }$$

(37) $$\epsilon_{yx}=\frac{F_{2}L^{3}cos\theta sin\theta }{12E_{s}I\left(2a+Lcos\theta \right)}\left[1+6\frac{a}{L}+2{\left(\frac{a}{L}\right)}^{2}\frac{1}{cos\theta }+2{\left(\frac{a}{L}\right)}^{3}\frac{1}{sin\theta cos\theta }+\left(3.4+1.5v_{s}\right){\left(\frac{t}{L}\right)}^{2}\right]$$

Using the ratio of the two strains, the Poisson’s ratio $v_{y}$ for loading in the **y-direction** in normalized form can be calculated as follows:

(38) $$v_{y}=-\frac{\epsilon_{yx}}{\epsilon_{xx}}$$

(39) $$v_{y}=-\frac{\left(\frac{H}{L}-sin\theta \right)sin\theta }{\left(2\frac{a}{L}+cos\theta \right)cos\theta }\left\{\frac{\left[1+6\frac{a}{L}+2{\left(\frac{a}{L}\right)}^{2}\frac{1}{cos\theta }+2{\left(\frac{a}{L}\right)}^{3}\frac{1}{sin\theta cos\theta }+\left(3.4+1.5v_{s}\right){\left(\frac{t}{L}\right)}^{2}\right]}{\left[1+6\frac{a}{L}+6{\left(\frac{a}{L}\right)}^{2}\frac{1}{cos\theta }+8{\left(\frac{a}{L}\right)}^{3}\frac{1}{cos^{2}\theta }+\left(2.4+1.5v_{s}+\frac{1}{\tan^{2}\theta }+\frac{H}{L}\frac{1}{cos^{2}\theta }\right){\left(\frac{t}{L}\right)}^{2}\right]}\right\}$$

Young’s modulus $E_{y}$ can be calculated using the stress $\sigma_{yy}$ and strain $\epsilon_{yy}$ in the **y-direction:**

(40) $$E_{y}=\frac{\sigma_{yy}}{\epsilon_{yy}}$$

(41) $$E_{y}=E_{s}\;{\left(\frac{t}{L}\right)}^{3}\frac{\left(\frac{H}{L}-sin\theta \right)}{\left(2\frac{a}{L}+cos\theta \right)cos^{2}\theta }\left[\frac{1}{\left[1+6\frac{a}{L}+6{\left(\frac{a}{L}\right)}^{2}\frac{1}{cos\theta }+8{\left(\frac{a}{L}\right)}^{3}\frac{1}{cos^{2}\theta }+\left(2.4+1.5v_{s}+\frac{1}{\tan^{2}\theta }+\frac{H}{L}\frac{1}{cos^{2}\theta }\right){\left(\frac{t}{L}\right)}^{2}\right]}\right]$$

## 4. Materials and Methods

### 4.1. Finite Element Analysis (FEA)

**Figure 4:**
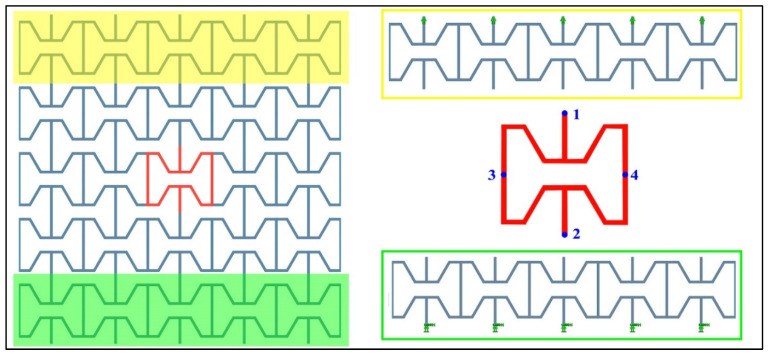
Boundary conditions for the FEA with points for measuring deflections.

For initial analysis, two commercial software were used (Solidworks simulation suite and Abaqus) to carry out the FEA of the structure. There was no variation in results for both software. For ease of modeling and the faster ability to perform multiple design studies, the Solidworks simulation suite was chosen for the FEA. A linear static study was performed to calculate the deflections of the structure. The shell-type element was used. A mesh size of 0.5 mm was used for all the studies conducted. For the FEA of such cellular structures, to avoid edge and size effects during deformation, it is recommended to use a minimum of four unit cells [12]. In this study, a 5 × 5-unit cell array was considered, and the deflections were measured at the center cell of the lattice. The center unit cell and boundary conditions used in the FEA are shown in Figure 4. The center unit cell was drawn in different colors and mentioned points to measure the deflection. The difference of these points divided by the length of the cell in the respective direction gave the strain in that direction. The ratio of the two strains provides us with Poisson’s ratio of the structure. The structure was set to be fixed in both directions at one end, and a load was applied at the other end, replicating the boundary conditions of a tensile test.

### 4.2. Samples Preparation and Experimental Procedures

**Figure 5:**
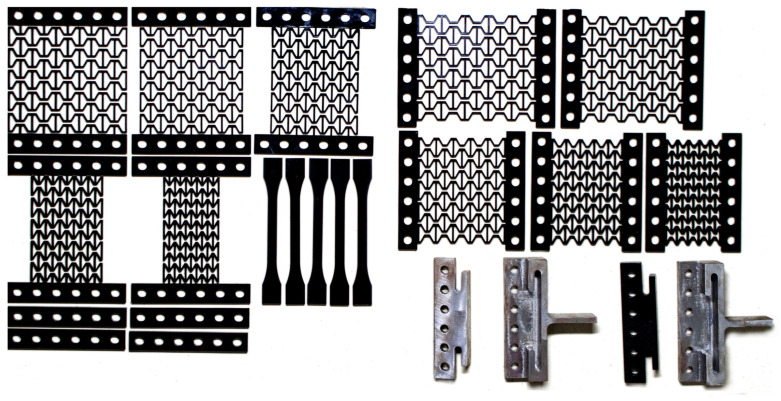
Laser-cut acrylic samples for testing loads in both the x and y-directions.

A total of 10 test samples were manufactured from 3.75 mm acrylic sheets. Thickness greater than that resulted in melting of the edges and sharp corners of the structure with the laser cutting. Half of the samples were for testing the properties in the x-direction, while the other half were for the y-direction. The parameters of the samples are shown in Table 1. All the samples were laser cut from the same sheet of acrylic to achieve the same material properties for each sample. In addition, five dog-bone-shaped samples were manufactured from the same sheet using the dimensions provided by the standard ISO 527-1 used for calculating the material properties of plastics. The laser-cut samples and the hinged support are shown in Figure 5.

In order to study the effect of the horizontal member, all the variables were fixed, except the length of the newly introduced strut a. The length is varied with 0.8 mm increments resulting in a/L values that range from 0.2 mm to 0.5 mm with 0.1 mm increments. Each sample was connected to a rectangular portion 120 mm in length and 20 mm in width. This portion contains six holes for 8 mm bolts to pass through. The samples with a = 0.8 mm (a/L = 0.1) showed clear manufacturing defects as it was too close to the edges of the connecting struts and difficult to manufacture with the existing setup. Thus, they were excluded, and experimental results were discarded for those samples.

**Figure 6:**
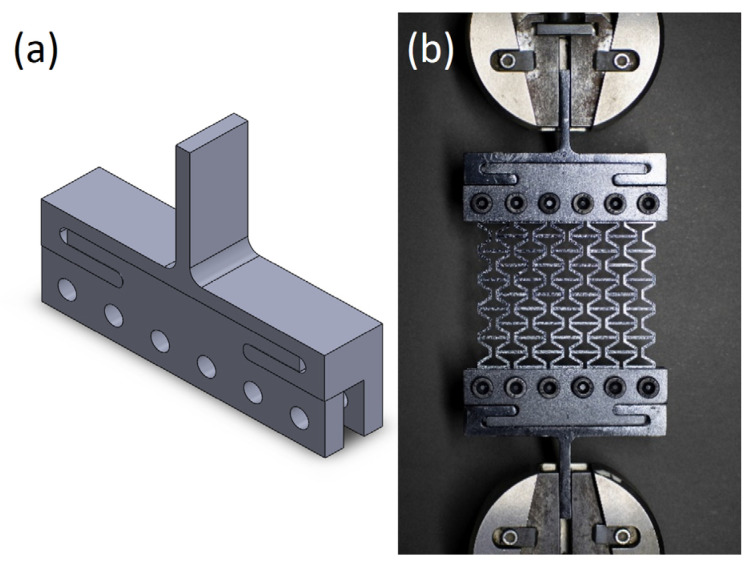
(**a**) Custom mount for holding the structure and (**b**) tension testing setup.

These 2D structures had to be tested with a tensile load, as the compressive load would result in buckling and out-of-plane deformation. A customized mild steel mount was designed to apply a uniform tension load to the structure, as shown in Figure 6. The mount was designed as a two-piece assembly that can hold the variable thickness of the sample for future work.

**Figure 7:**
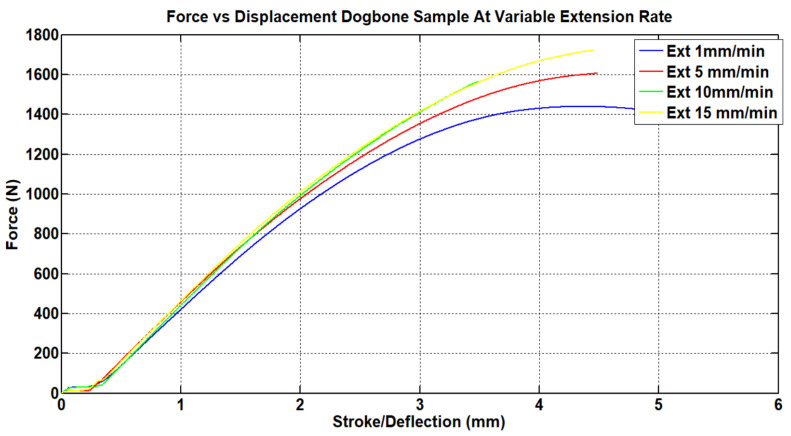
Load vs. displacement graph for acrylic dog-bone samples.

Tensile testing of the samples was performed using an Autograph AGS-J tabletop Universal Testing Machine (UTS). Tensile testing of the dog-bone samples was carried out at different extension rates according to ISO 527-1 to determine the Young’s Modulus of the material. The results of these tests can be seen in Figure 7. The strain rate is sensitive for polymers since the molecular mobility of the polymer chains becomes stiffer with the increment in strain rate [29]. The deflection rate for the modified re-entrant structure specimen was set at 10 mm/min, as this value shows less sensitivity to materials behavior.

The black color specimens were sprayed with a white dotted pattern to determine the deflections through image processing during the tensile tests. The experiment was filmed at 1080p using a DSLR camera at 30 frames per second. The deflection of the structure was analyzed by tracking the movement of each individual dot using a program, GOM Correlate [30].

## 5. Results and Discussion

**Figure 8:**
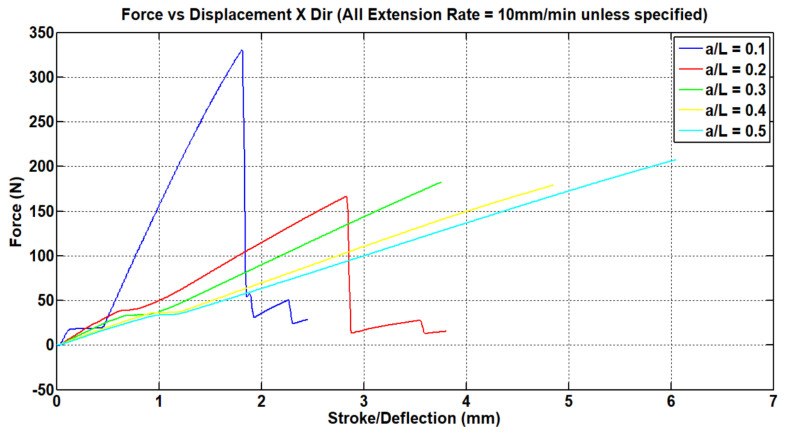
Force vs. displacement plot for loading in the x-direction.

The force vs. displacement results obtained for the structure under loading in the x- and y-directions can be seen in Figure 8. As we increase the values of ***a/L***, the structure becomes more compliant at the expense of failure force.

**Figure 9:**
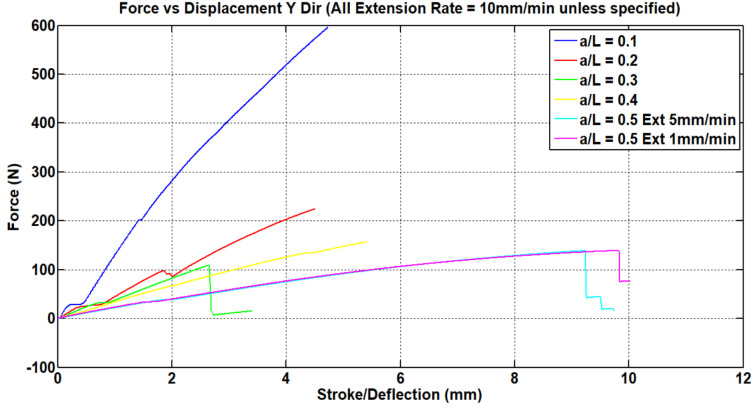
Force vs. displacement plot for loading in the y-direction.

**Figure 10:**
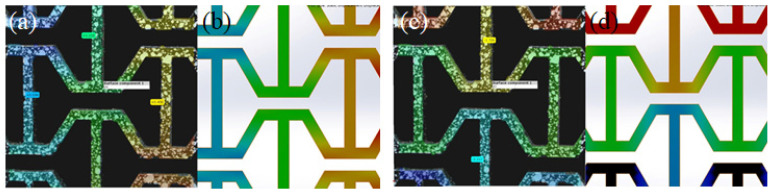
Deflection pattern between the tensile test and FEA for loading in (**a**,**b**) the x-direction and (**c**,**d**) y-direction.

In the next section, a detailed comparison of the values of Poisson’s ratios in both principal directions is given. However, looking at the test data from Figure 8 and Figure 9, we can see that the area under the graph increases by increasing the values of ***a/L***, suggesting that the structure can absorb more energy compared to the re-entrant honeycomb model. Figure 10 compares the deflections in both the x- and y-directions obtained by FEA and testing results when overlapped with the image processing software. The behavior of the structure was almost the same, exhibiting the auxetic nature of the structure, and the relative deflection pattern in both cases was similar.

### 5.1. Effect on NPR with Different UC Parameters

#### 5.1.1. Loading in the X-Direction

**Figure 11:**
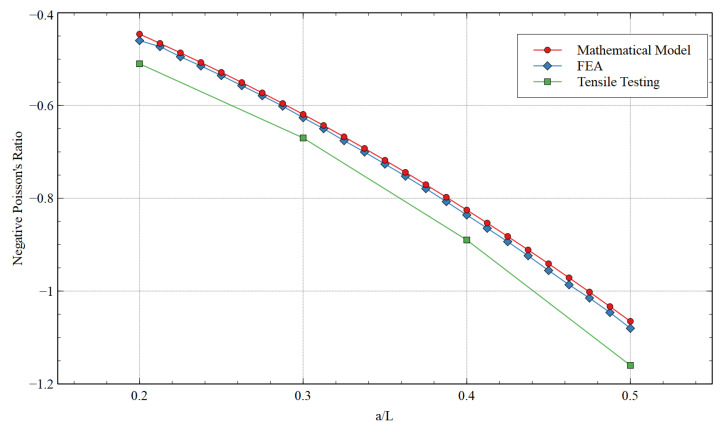
Poisson’s ratio obtained via a mathematical model, FEA, and tensile testing for loading in the x-direction.

The comparison between the values of NPR obtained using the mathematical model, FEA, and the experimental testing is shown in Table 2 and Figure 11. The NPR obtained by all three methods exhibited close values and followed the same trend with approximately constant error margins. It is interesting to note that the modified structure’s auxetic behavior increased with the added strut value. When the ***a/L*** ratio was increased from 0.2 to 0.5, the tensile test showed that the NPR enhanced by 127%, which is a significant improvement. The additional strut provides greater flexibility to the re-entrant strut to enhance the Poisson’s ratio in the x-direction.

**Figure 12:**
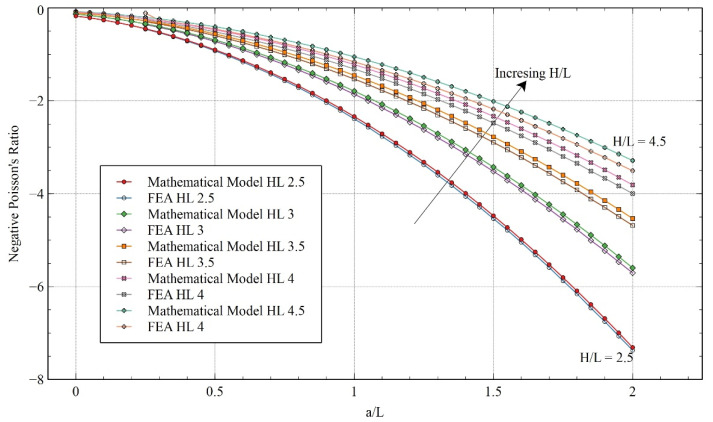
Poisson’s ratio obtained from the FEA and mathematical model for varying ***H/L*** for x-direction loading.

Once the FEA model was validated with the experimental results, a more detailed comparison between the mathematical model and FEA was conducted by varying the other parameters of the unit cell. In Figure 12, the comparison is performed by keeping the angle θ constant at 60° while the value of ***H/L*** is varied. Even in the extended range, the trends of both calculations followed the same pattern. Furthermore, it can be observed that by reducing the value of ***H/L***, the value of NPR increased. Reducing the value of ***H/L*** means a reduction in the mass or relative density of the structure, which can be favorable in certain scenarios. Setting the value of ***a/L*** equal to zero results in the structure becoming a standard re-entrant honeycomb structure. Comparing the proposed structure with the re-entrant honeycomb model, we can see a significant increase in auxeticity for varying values of ***H/L***.

**Figure 13:**
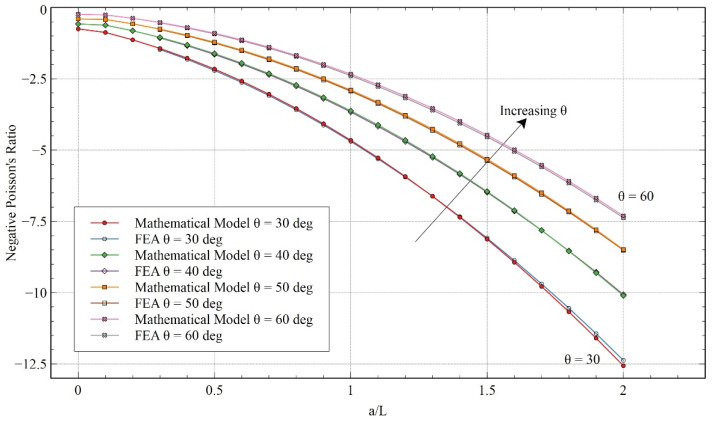
Poisson’s ratio obtained from the mathematical model and FEA for varying θ for x-direction loading.

On the other hand, in Figure 13, ***H/L*** is kept constant, and the angle θ is varied for an extended range of ***a/L***. The results suggested that decreasing the re-entrant angle resulted in an enhancement of NPR. The mathematical model started deviating from the FEA when approaching higher re-entrant angles, but the trend remained the same, and the variation remained low. Previously significant gains in NPR can be seen compared to the re-entrant honeycomb model across the board.

#### 5.1.2. Loading in the Y-Direction

**Figure 14:**
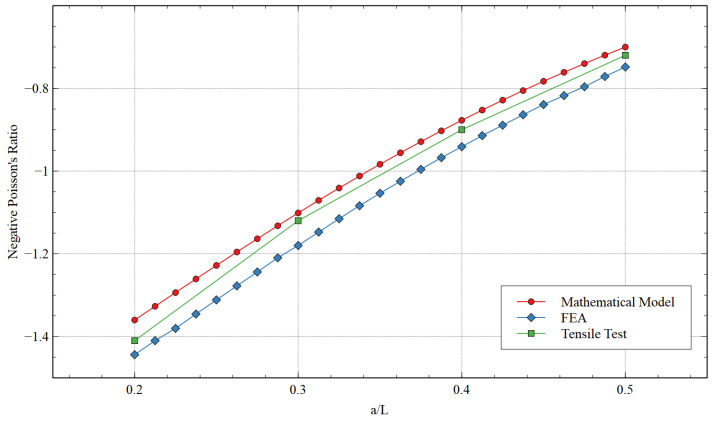
Poisson’s ratio obtained via the mathematical model, FEA, and tensile testing for loading in the y-direction.

The comparison between the values of NPR obtained using the mathematical model, FEA, and the experimental testing is shown in Table 3 and Figure 14. A sharp contrast can be observed here when compared to the NPR values when loading was in the x-direction. The increment in ***a/L*** showed a counter impact on NPR. Interestingly, the absolute values of NPR when the load was applied in the y-direction were high. During the tensile test, when ***a/L*** was 0.2, the NPR when loading in the x- and y-directions was −0.51 and −1.41, respectively. Therefore, high NPR can be achieved if the structure is oriented to apply a unidirectional load in the y-direction. However, when the a/L value was 0.4, the NPR value was approximately the same during tensile testing (NPRs when loading in the x- and y-directions were −0.89 and −0.9, respectively). This was the limiting ratio for a/L; after that, when the ***a/L*** equal to 0.5 was analyzed during tensile testing, the NPR was higher for the x-direction rather than in y-direction loading (NPRs when loading in the x- and y-directions were −1.16 and −0.72, respectively). These results are intriguing, as the orthotropic unit cell can exhibit isotropic behavior at certain unit cell parameters. Here, in this case, it was θ constant at 60 degrees and ***a/L*** ≃ 0.4.

**Figure 15:**
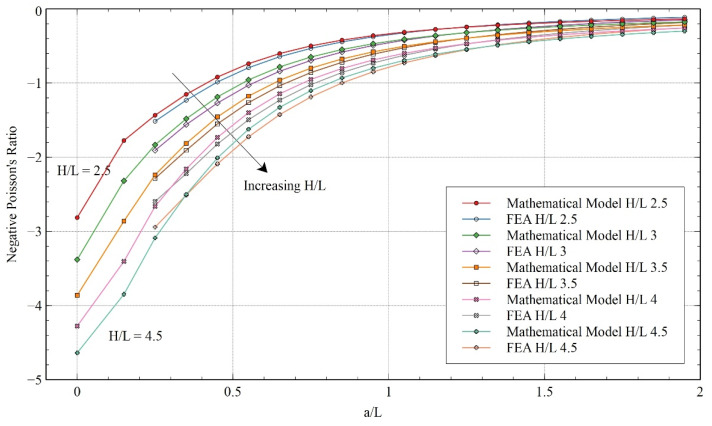
Comparison of Poisson’s ratio obtained from the FEA and mathematical model for varying ***H/L*** for y-direction loading.

**Figure 16:**
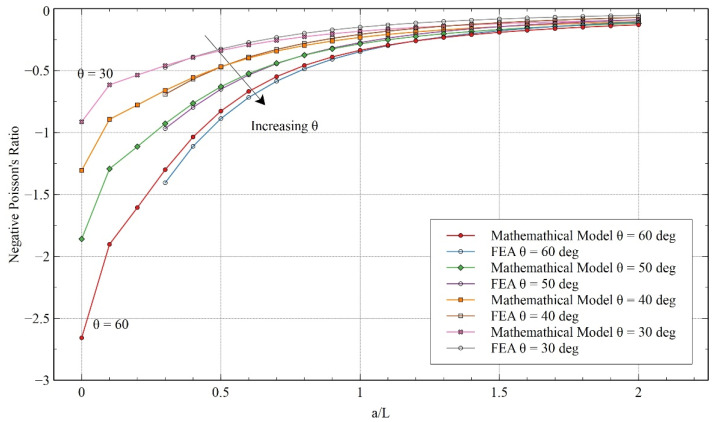
Comparison of Poisson’s ratio obtained from the FEA and mathematical model for varying angle θ for y-direction loading.

Figure 15 and Figure 16 give a detailed comparison of NPR between the mathematical model and FEA for different ***H/L*** and re-entrant angles. The results gave an insight into the effects of these variables on NPR. As observed before, the trends exhibited by the mathematical model corroborate the trend obtained from FEA. In both cases, it was observed that NPR decreased as the length of the horizontal member increased. The NPR lost auxeticity rapidly for smaller values of ***a/L*** but started leveling out at the extreme values of ***a/L***.

Contrary to x-direction loading, here it can be observed that the NPR increased with the value of ***H/L*** (see the arrow direction in Figure 15). Similarly, it can be noticed that NPR increases as the re-entrant angle decreases (see the arrow direction in Figure 16). Comparing the structure with the re-entrant honeycomb model, we can see that, in this specific case, better NPR can be achieved using the re-entrant honeycomb model.

#### 5.1.3. Comparison of NPR

**Figure 17:**
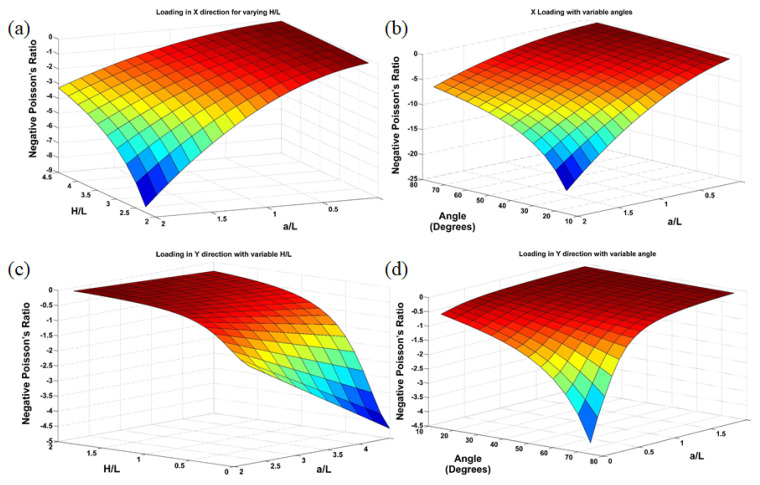
NPR for different values of H/L, ***a/L***, and re-entrant angle for (**a**,**b**) x-direction loading and (**c**,**d**) y-direction loading.

The combined effect on NPR calculated using the mathematical model for the variation of ***H/L***, ***a/L***, and re-entrant angle for both loading directions is shown in Figure 17. In general, when the loading was applied in the x-direction, the NPR was improved with low values of ***H/L***, re-entrant angle, and higher values of ***a/L***. This was because the added strut ***a*** is in the x-direction, and the lower value was favorable for the re-entrant strut to improve NPR. In contrast to the x-direction loading, the NPR showed mixed behavior when the loading was applied in the y-direction. NPR improved with either low values of ***H/L*** and high values of ***a/L*** or high values of re-entrant angle and low value of ***a/L***.

### 5.2. Young’s Modulus of the Structure

**Figure 18:**
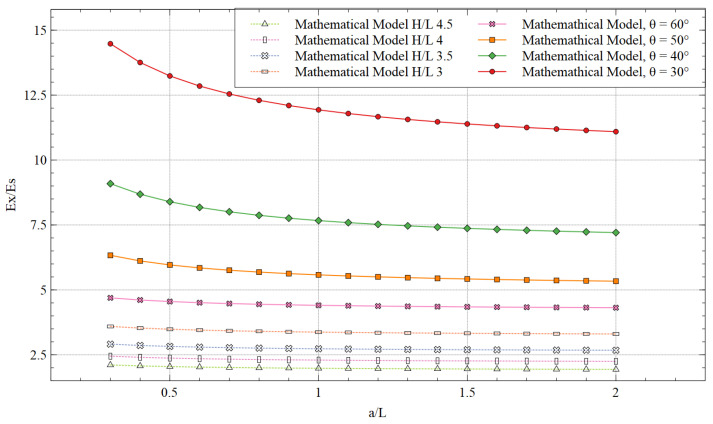
Young’s modulus in the x-direction for different values of ***H/L*** and re-entrant angle.

**Figure 19:**
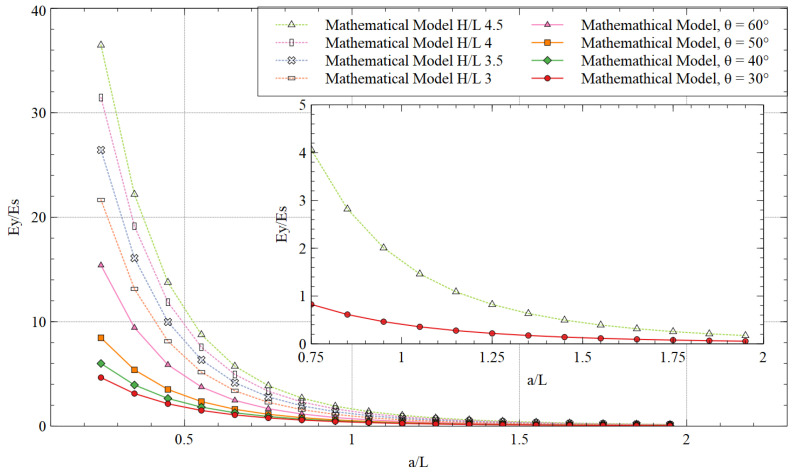
Young’s modulus in they-direction for different values of ***H/L*** and re-entrant angle (inset shows a portion of the extreme values).

The ratios of Young’s modulus for respective loading direction and the solid material of the structure, calculated by the mathematical model, are shown in Figure 18 (***θ*** = 60°) and Figure 19 (***H/L*** is 2.5). There was a significant improvement in NPR in both loading directions. The unit cell improved the strength of the material by adding the strut and making it stiffer. For x-direction loading, increasing the length of additional strut ***a*** or ratio ***a/L*** did not show a considerable variation in enhancement in the strength. However, lower values of ***a/L*** showed a stiffer behavior of the structure. The additional strut ***a*** is parallel along the x-direction, and thus the bending effect was low when the load was applied in the same direction. As a result, the variation in Ex/Es was also low.

On the other hand, the strut is perpendicular to the y-direction and can be subjected to extensive bending during the tensile load. Therefore, significant variation was observed with the increasing ***a/L*** ratio or varying re-entrant angle. However, the strength in the y-direction is significantly higher than in the x-direction. In fact, the structure showed stiffness in the y-direction for smaller values of ***a/L***, which diminished with the ***a/L*** ratio being approximately more than 1.25. Nonetheless, a tradeoff for the strength can be made in both directions depending on the application of the structure.

### 5.3. Effect of Relative Density on NPR

**Figure 20:**
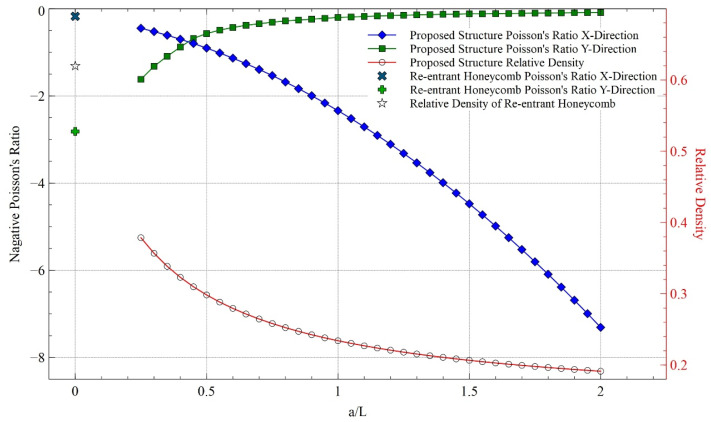
Relative density and NPR for loading in both directions.

The relative density and NPR of the structure for different values of ***a/L*** during loading in both directions (***H/L*** is 2.5 and ***θ*** = 60°), calculated from the mathematical model, is shown in Figure 20. The relative density is the ratio of the volumes of the structure to the total volume it occupies if it is solid. It can be seen that with the increasing length of additional strut **a**, the relative density decreases while the NPR significantly increases in x-direction loading. There is a reverse effect on y-direction loading, but the variation is not high. It can be concluded that for a given thickness of the structure inside a fixed volume, higher NPR is possible while decreasing the overall mass of the structure for loading in the x-direction only. For comparison, the relative density and NPR for both directional loading for a standard re-entrant honeycomb structure are also shown in Figure 20. It can be observed that added strut highly improved the relative density and significantly improved NPR in x-direction loading.

## 6. Conclusions

The modified re-entrant honeycomb auxetic structure with enhanced directional properties is proposed and analyzed through a mathematical model, FEA, and experimental test. The modification was made by adding a strut connecting the vertical and re-entrant members of the re-entrant honeycomb unit cell. A detailed analytical model has been derived that considers bending, shear, and axial deformations to calculate Poisson’s ratio and other properties. Tensile tests were performed on laser−cut samples to validate the mathematical and FEA models. All three methods showed close agreement with the proposed structure’s negative Poisson’s ratio (NPR). The structure was analyzed by applying the loading in two principal directions on the doubly symmetrical unit cell. When the loading was applied in the x-direction, the NPR was improved with lower values of ***H/L*** and re-entrant angle, and higher values of ***a/L***. On the other hand, when the loading is applied in the y-direction, the NPR showed mixed behavior. NPR improved with a combination of either low values of ***H/L*** and high values of ***a/L*** or high values of re-entrant angle and low values of ***a/L***.

For a range of values of ‘***a/L***’, the relative density of the structure decreases with the increasing value of ‘***a***’. A lower relative density suggested that the structure utilized the occupied volume more effectively, meaning that it could provide a better NPR-to-mass ratio. To relate the relative density to NPR, the value of ‘***a***’ should be increased to obtain better relative density and an increase in Poisson’s ratio in the x-direction at the cost of a decrease in Poisson’s ratio in the y-direction. To improve its properties, the structure can be further modified by increasing the thickness of struts or adding cross-link members, making it a hybrid structure at the cost of losing NPR.

The proposed structure could have many applications in areas that require a greater NPR. For example, filters, energy absorption, lightweight structures, etc. It would also be interesting to analyze the bi−directional loading on the proposed auxetic cellular structure to analyze the combined loading effect.

## Figures and Tables

**Table 1:** Dimensions of the test samples.

L	H	t	b	a	θ	H/L	t/L	a/L	Young’s Modulus $\;E_{s}$
mm	mm	mm	mm	mm	Degrees				MPa
8	20	1.75	3.75	1.6 2.4 3.2 4.0	60	2.25	0.218	0.2 0.3 0.4 0.5	1050

**Table 2:** Comparison of Poisson’s ratio obtained from the mathematical model, FEA, and tensile testing for loading in the x-direction.

*a*/*L*	Mathematical Model	FEA	Tensile Test
0.2	−0.45	−0.46	−0.51
0.3	−0.61	−0.62	−0.67
0.4	−0.82	−0.83	−0.89
0.5	−1.06	−1.08	−1.16

**Table 3:** Poisson’s ratio obtained from the mathematical model, FEA, and tensile testing for loading in the y-direction.

*a*/*L*	Mathematical Model	FEA	Tensile Test
0.2	−1.36	−1.46	−1.41
0.3	−1.1	−1.18	−1.12
0.4	−0.88	−0.94	−0.9
0.5	−0.7	−0.75	−0.72

## Data Availability

There is no external data available with this work. All data, models, and code generated or used during the study appear in the submitted article.
